# TGF-β1/Smad Signalling in Proliferative Glomerulonephritis Associated with Autoimmune Diseases

**DOI:** 10.31138/mjr.33.2.176

**Published:** 2022-06-30

**Authors:** Aglaia Chalkia, Harikleia Gakiopoulou, Irini Theochari, Periklis G. Foukas, Dimitrios Vassilopoulos, Dimitrios Petras

**Affiliations:** 1 Nephrology Department, Hippokration General Hospital, Athens, Greece,; 2 1^st^ Department of Pathology, National and Kapodistrian University of Athens, School of Medicine, Athens, Greece,; 3 2^nd^ Department of Pathology, National and Kapodistrian University of Athens, School of Medicine, Athens, Attikon University Hospital, Athens, Greece,; 4 2^nd^ Department of Medicine and Laboratory, Clinical Immunology - Rheumatology Unit, National and Kapodistrian University of Athens, School of Medicine, Athens, Greece

**Keywords:** proliferative glomerulonephritis, TGF-β1/Smad signalling, fibrosis, inflammation

## Abstract

Glomerulonephritis is a common cause of chronic kidney disease, which has emerged as a major cause of end-stage renal disease. Autoimmune diseases, such as Systemic Lupus Erythematosus (SLE) and ANCA-associated vasculitis (AAV) are often associated with proliferative glomerulonephritis. Transforming growth factor-β1 (TGF-β1) is a cytokine with pleiotropic effects in chronic renal diseases, based on in vivo and in vitro studies. The Smad-dependent signalling pathway plays an important role in the regulation of renal fibrosis (excessive production of extracellular matrix [ECM]) and inflammation. However, clinical trials targeting TGF-β1 have presented disappointing results, suggesting that the downstream signalling is quite complex. The diversity of the effects may associate with the interactions between TGF-β1 signalling and other downstream signalling, as well as the different cellular responses, which TGF-β1 promotes. Recently, macrophage chemoattract and epigenetic effects have also been identified as new mechanisms, wherefore TGF-β1/Smad signalling mediates renal injury. This review provides an overview of the role of TGF-β1/Smad signalling pathway from in vivo and in vitro studies in the pathogenesis of glomerulonephritis and particularly in proliferative glomerulonephritis, which is associated with autoimmune diseases.

## INTRODUCTION

The proliferative glomerulonephritis (GN) is characterised by glomerular infiltration by inflammatory cells, such as neutrophils and macrophages, and/or proliferation of resident glomerular cells. These cells may induce thrombosis, necrosis, and crescent formation, resulting in rapidly progressive GN.^1^ The renal injury includes humoral (B cell activation, plasma cells) and/or cellular (T-helper cells, mononuclear inflammatory cells) immune response. ANCA-associated vasculitis (AAV) and Systemic Lupus Erythematosus (SLE), and more rarely other autoimmune diseases, such as primary Sjogren’s syndrome (pSS), Rheumatoid arthritis (RA), Scleroderma (SS), are associated with proliferative GN.

Transforming growth factor-beta 1 (TGF-β1) is a multi-functional cytokine that regulates cell proliferation, differentiation, apoptosis and adhesion. Recent studies have also shown new mechanisms, whereby TGF-β can mediate renal injury, such as macrophage chemoattractant and epigenetic effects. ^2,3^ Although, the role of TGF-β1 in the pathogenesis of glomerulosclerosis and renal fibrosis in patients with podocytopathies, such as focal and segmental glomerulosclerosis (FSGS) has been demonstrated, the signalling is also activated in proliferative GN, and correlates with the severity of inflammation. ^4,5^

In AAV, probably after exposure to infectious agent, TGF-β1 and interleukin (IL)-6 are released from dendritic cells and induce differentiation of naïve T cells into T helper 17 (Th17) cells. Th17 produce IL-17 and stimulate macrophages to produce tumour necrosis factor (TNF)-a and IL-1β, which act as major priming factors to neutrophils.^6,7^ Therefore, neutrophils are activated and present the MPO or PR3 target antigens. In lupus nephritis, the immune complex deposition in glomeruli can activate inflammatory response, which can recruit inflammatory cells and activate the glomerular cells. Increased levels of TGF-β1 have been detected in lupus renal tissue, and a positive correlation with histological activity has been reported. ^8,9^ Recently, in SLE new targets of autoantibodies have been confirmed to interact with TGF-β1 signalling, such as Smad2 and Smad5 protein.^10^

Interestingly, recent studies have demonstrated the involvement of TGF-β/Smad signalling in pSS salivary glands (SG) as a mediator of the epithelial-mesenchymal transition (EMT) activation. Furthermore, pSS SGs biopsy specimens were characterised by an elevated expression of TGF-β1 in the glandular epithelium, and TGF-β1, pSMAD2/3, and SMAD4 proteins were widely expressed in the pSS tissue in patients.^11^ In RA TGF-β/Smad3 signalling was markedly activated in synovial tissues, which was associated with the loss of Smad7, and enhanced Th17 and Th1 immune response.^12^ TGF-β signalling also participates in the progression of fibrosis in SS. High levels of TGF-β1 and its regulated genes have been detected in skin biopsies and were positively correlated with the severity of SS.^13^

In this review, we present an image of the role of TGF-β signalling in the pathogenesis of glomerular injury, especially in proliferative GN associated with autoimmune diseases.

### TGF-β1 and Smad pathway

While in the normal human kidney TGF-β1 is negligibly expressed, under pathological circumstances it is synthesised by many renal cells and contributes to glomerular filtration barrier alteration, fibrosis, sclerosis, and tubule degeneration.^11^ Many factors, such as high level of glucose, oxidate stress, and cytokines can stimulate transcription of the TGF-β1 gene. Furthermore, activated T and B cells, macrophages, neutrophils, immature hematopoietic cells, and dendritic cells also produce TGF-β1 and/or are sensitive to its effects.^14^

**Figure 1.**
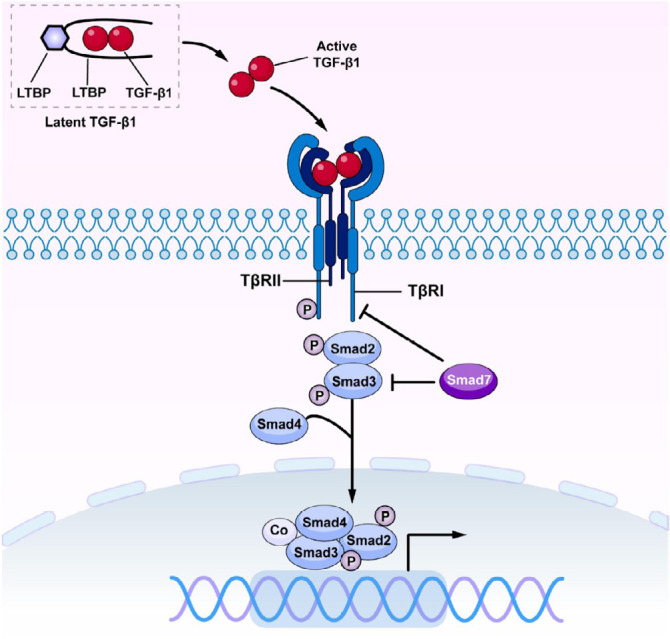
TGF-β1 binds TβRII and activated Smad2 and Smad3, resulting in formation of a complex with Smad4. The Smad2/3/4 complex then translates into the nucleus and binds to the target genes to induce fibrosis and inflammation. TGF-β, transforming growth factor β; TβRI, TGF-β receptor type I; TβRII, TGF-β receptor type II (modified^24^)

There are three isoforms of TGF-β present in mammals. Among them, TGF-β1 has reported as an important and crucial mediator in the pathogenesis of progressive renal fibrosis. ^15–17^ TGF-β1 is synthesised as a part of a biologically inactive complex and after proteolytic cleavage becomes available to bind to receptor complexes (TGFβRI) (**Figure 1**). Then, the canonical Smad signalling is activated.^18^ The Smad2 and Smad3 are phosphorylated and their complex translocate into the nucleus to modulate the transcription levels of target genes.^19–21^ Therefore, TGF-β1 induces transcription of several miRNA species, with some miRNAs showing profibrotic and other antifibrotic effects.^21–23^ Smad7 can compete with Smad2 and Smad3 for binding to activated TGFβR1 and thus serves as negative feedback inhibitor of TGF-β1/Smad canonical signalling.

Clinical trials in diabetic nephropathy, septic acute kidney injury and focal segmental glomerulosclerosis have examined the direct targeting of TGF-β1 with disappointing results, highlighting the diversity and complexity of TGF-β1 signalling in renal fibrosis and inflammation. ^24^ However, targeting downstream signalling by specifically inhibiting or overexpressing Smad3-dependent non-coding RNAs or rebalancing Smad3/Smad7 may be a better approach.^24^

### Renal Fibrosis and TGF-β1

It is well accepted that TGF-β1/Smad signalling is a major pathway for renal fibrosis, synthetizing extracellular matrix (ECM) protein in both the glomerulus (glomerulosclerosis) and the tubulointerstitial tissue (interstitial fibrosis). Initials studies focused on this growth factor’s effects on fibroblasts via promoting activation of myofibroblasts.^25^ However, its role also includes proliferation, differentiation, hypertrophy, apoptosis, angiogenesis, cell cycle control, chemotaxis, and haematopoiesis. In experimental and human kidney diseases with renal fibrosis, regardless the initial cause of chronic kidney disease (CKD), TGF-β1 signalling is activated, such as diabetic nephropathy,^26–28^ obstructive kidney disease,^29^ ⅚ nephrectomy,^30^ hypertensive nephropathy,^31^ and glomerular diseases (IgA nephropathy, FSGS, lupus nephritis, crescentic GN). Transgenic mice with increased circulating levels of TGF-β1 developed glomerulosclerosis and those with increased tubular production of TGF-β1 developed tubulointerstitial fibrosis in the absence of any additional injury.^32^ Furthermore, the role of Smad3 in renal fibrosis is supported, because genetic inhibition of Smad3 reduced ECM in unilateral ureteral obstruction (UUO).^33^

### Renal Inflammation and TGF-β1

The role of TGF-β1 in inflammation after renal injury is more complex. It is generally accepted that renal inflammation serves as the initial event of renal fibrosis in CKD, and the persistent activation of inflammatory processes promotes fibrogenous responses. Despite this proinflammatory role, TGF-β1 also possesses anti-inflammatory responses. Firstly, this was demonstrated, because mice that lack TGF-β1 develop uncontrollable systemic inflammation and die 3 weeks after birth.^34^ Furthermore, the protective role of TGF-β1 was reported in immune-mediated kidney disease in transgenic mice by administering the sheep anti-mouse glomerular basement membrane (GBM) antibody. Interestingly, mice with experimental crescentic GN, which had increased levels of latent, but not active, TGF-β1 in plasma and kidney tissue, and upregulation of renal Smad7 in keratinocytes, preserved renal function and were protected against renal fibrosis.^35^ Likewise, a recent study showed that it is possible to dissociate the fibrotic effect of TGF-β1 from its anti-inflammatory effect, by preventing the cross-talk interaction with the Wnt/β-catenin pathway.^36^

### TGF-β1 signalling and inflammatory cells (**Figure 2**)

#### T cells

There is increasing evidence supporting the role of TGF-β1 in inflammation, both pro- and anti-inflammatory effect. On one hand, TGF-β1 can induce activation of Foxp3+, regulatory T cell subset (Treg), which suppresses renal injury and on the other hand can induce T cell differentiation to T17 subtype (Th17), which plays a significant role in inflammation in some forms of GN.^37^ These cells are called Th17 cells because they produce the interleukin (IL)-17 cytokine. Th17 cells also promote autoimmune anti-MPO-mediated GN through the secretion of IL-17a.

**Figure 2.**
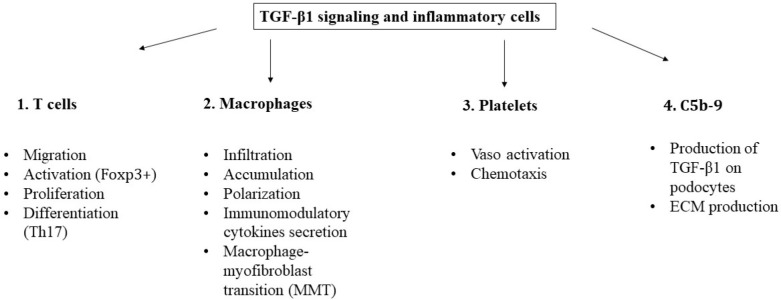
TGF-β1 signalling and inflammatory cells.

#### Macrophages

Recent investigations have also explored the role of TGF-β signalling in macrophage differentiation during inflammation**.** Macrophage-myofibroblast transition (MMT) is a newly identified phenomenon driven by TGF-β1 signalling as a direct mechanism of macrophage for promoting myofibroblast generation under unresolved renal inflammation.^38^ The macrophage infiltration has been shown to correlate with the severity of renal injury.^39^ Macrophages are divided into two types: the proinflammatory M1-type (classically activated) and the anti-inflammatory M2-type (alternatively activated). In acute kidney injury, in the early phase, there is recruitment of macrophage, which polarized into M1-type by various inflammatory mediators, including Th17 cells. This differentiation is induced from TGF-β1, indicating the role as a macrophage chemoattractant. Subsequently, in the repair phase of acute kidney injury TGF-β1 signalling can induce a M2 macrophage polarization, which may suppress inflammation, but the uncontrolled activation can promote fibrosis. However, selective deletion of TGF-β1 from macrophages did not alter fibrosis in animal model.^40,41^

#### Platelets

Platelets secrete vasoactive, chemotactic, and mitogenic substances that interact with mediators generated by renal resident or inflammatory cells and could contribute to glomerular injury. It is believed that growth factors, such as TGF-β1, are released from platelets and play an important role in this process.

#### C5b-9 (membrane attack complex)

The sublytic effects of C5b-9 on podocytes not only lead to proteinuria (mainly in membranous glomerulopathy) and produce hydrogen peroxide, but also increase the expression of TGF-β1 and its receptors, leading to overproduction of extracellular matrix resulting in GBM thickening.^42^

### TGF-β1 mediated renal pathology

**Figure 3.**
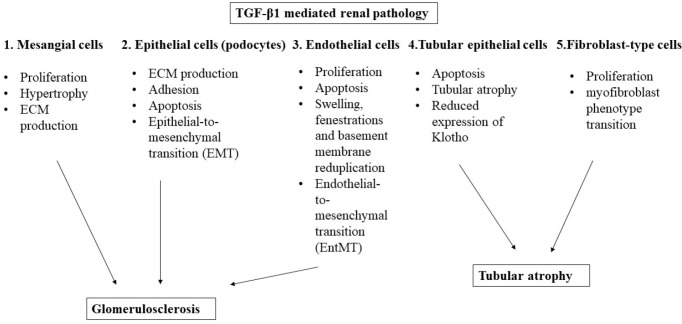
TGF-β1 mediated renal pathology.

It has been demonstrated that TGF-β1 affects various renal cells, including mesangial, endothelial cells, epithelial cells (podocytes), and tubular epithelial cells. (**Figure 3**)

#### Mesangial cells

TGF-β1 stimulates the mesangial cells to synthetizing type I, III and IV collagen, laminin, fibronectin and heparan sulphate proteoglycans, as well as plays a role in mesangial hypertrophy. Therefore, TGF-β1 is a major contributor to glomerular ECM accumulation by stimulating mesangial cells.^43^

#### Epithelial cells (podocytes)

The podocytes play a critical role in maintaining the glomerular integrity and function. They also synthesise most components of the GBM. It has been shown that podocytes with highly expression of TGF-β1 lead to apoptosis. In addition, in these damaged podocytes Smad7 expression is strongly expressed.^44,45^ Furthermore, in vitro experiments have shown that TGF-β1 induces epithelial-to-mesenchymal transition (EMT) after podocyte injury.^46^

#### Endothelial cells

It has also been demonstrated that TGF-β1 is a central inducer of endothelial-to-mesenchymal transition (EntMT) of these cells, cell proliferation and apoptosis. These changes contribute to proteinuria, inflammation, and glomerulosclerosis. A recent study reported that endothelial cells react differently in Smad3 deletion compared with podocytes. Hence, changes to glomerular endothelial cells, such as swelling, fenestrations and basement membrane reduplication were Smad3 dependent.^47^

#### Tubular epithelial cells

The expression of TGF-β1 has also been associated with tubular apoptosis.^48^ However, recently it has been shown a beneficial effect of TGF-β1 in proximal tubule. One study in animal CKD model reported that selective deletion of proximal tubular TβRII deteriorated tubular apoptosis. This may be reported in part through reduced β-catenin activity or through beneficial effect of TGF-β1 on autophagy. Likewise, recent evidence showed that TGF-β1 signalling reduced expression of Klotho, which is produced in proximal renal tubule.^49^

#### Fibroblast-type cells

TGF-β1 signalling can also induce proliferation and myofibroblast transition to intrinsic renal fibroblast-type cells, including interstitial fibroblasts and pericytes. It has also been demonstrated that damaged tubular epithelial cells secrete TGF-β1, which induce myofibroblast transition to the adjacent pericytes. ^50^

### Expression of TGF-β1/Smad signalling in Proliferative glomerulonephritis

**Figure 4.**
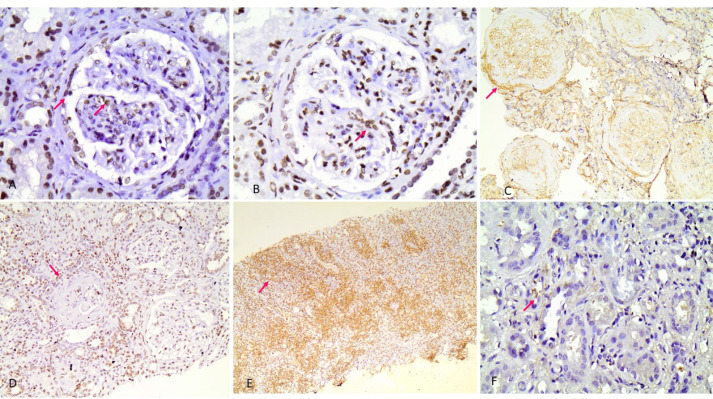
A, B, C Lupus nephritis (arrows indicating the positive immunostaining). Immunohistochemical evaluation of pSmad3 (**A**), Smad7 (**B**), TGF-β1 (**C**). D, E, F ANCA-associated glomerulonephritis (arrows indicating the positive immunostaining). Immunohistochemical evaluation of pSmad3 (**D**), Smad7 (**E**), TGF-β1 (**F**).^53^

TGF-β1/Smad signalling is activated and highly expressed in progressive forms of human kidney disease (**Figure 4**).^51–53^ TGF-β1 has been reported to serve as a critical mediator in the pathogenesis of glomerulosclerosis in glomerular diseases, including lupus nephritis and crescentic GN.^52–54^ Although the upregulation of TGF-β1 has been proved its role in the pathogenesis of renal fibrosis, glomerular immunoreactivity for TGF-β1 isoforms is also correlated with the severity of proliferative lesions, especially in lupus nephritis.^54^ Significant upregulation of the three TGF-β1 isoforms as well as TGFβRI and TGFβRII have been demonstrated in the glomerular, tubular, and interstitial area in kidney diseases. Furthermore, the urinary TGF-β1 level is increased and correlated with the severity of tubular-interstitial fibrosis.^55^ In TGF-β1 transgenic mice, an acute and massive increase in plasma levels of TGF-β1 results in severe GN with crescents.^48–49^ TGF-β1 expression is also strong in the cellular crescents.^56^ In disorders with abnormal glomerular and tubulointerstitial matrix accumulation, including crescentic GN and diffuse proliferative lupus nephritis, were noted significant increases in the immunoreactivity of all three TGF-β isoforms in glomeruli (p<0.025) and tubulointerstitium (p < 0.025).^57^

The effect of Smad pathway in proliferative (including pauci-immune GN and lupus nephritis III, IV) and non-proliferative GN was examined and demonstrated that pSmad 2/3 was increased in all glomerular cells and was positively correlated with serum creatinine level and interstitial inflammation in both GN.^58^ A recent study evaluated the role of Smad3 as an important mediator of glomerulosclerosis and interstitial fibrosis in a model of proliferative crescentic GN. SMAD3−/− mice had only transient proteinuria, and the glomerular endothelium demonstrated transient injury, which was temporally correlated with proteinuria.^47^ Interestingly, the effect of Smad3 deletion was different between the glomerular cells.

**Table 1.** Selected studies demonstrating expression of TGF-β1/Smad signalling in autoimmune disease- associated glomerulonephritis.

**Ref**	**Disease model**	**Molecule**	**Urine/plasma/tissue**	**Correlation with histopathological characteristics**	**Correlation with clinical characteristics**
35	Crescentic GN mouse model	Latent TGF-β1	Plasma/renal tissue	Protection against crescent formations and T cells and macrophage infiltration	Preservation of renal function Reduction of proteinuria
49	Crescentic GN mouse model	Smad3	Renal tissue	Glomerulosclerosis, Interstitial fibrosis Glomerular endothelial cells (loss of fenestrations, swelling, and basement membrane reduplication)	Proteinuria
57	GN with proteinuria (including lupus nephritis) human model	TGF-β1	Renal tissue/urine/plasma	Tubular epithelial cells Interstitial expression Lower expression in glomeruli Interstitial inflammation/fibrosis Tubular atrophy	Proteinuria
58	Crescentic GN mouse model	TGF-β1	Renal tissue	Cellular/fibrous cellular crescents	N/A
60	GN human model	TGF-β1 TGF-βLAP	Renal tissue	Increase of mesangial matrix and matrix components of GBM Immune deposits in glomeruli	serum N/A
61	Crescentic GN mouse model	Smad7 gene therapy	Renal tissue	Attenuation of renal fibrosis and inflammation Inhibition of interstitial mononuclear cell infiltration, crescent formations and glomerulosclerosis	Reduction of proteinuria Improvement of renal function
62	Lupus nephritis human model	Mir-150	Renal tissue	Glomerulus sclerosis Fibrous crescents Tubular atrophy Interstitial fibrosis	Chronicity index [CI]≥4
63	Lupus nephritis mouse model and renal glomerular endothelial cells	Mir-183 TGF-βRI	Renal tissue	Mir-23 is reduced in LN Overexpression of Mir-23 inhibits inflammatory cell infiltration and renal fibrosis TGF-βR1 highly expressed in LN	Overexpression of Mir-23 reduced proteinuria TGF-βRI: renal fibrosis
53	GN (including crescentic and Lupus nephritis) human model	TGF-β1 SMAd7 pSMad3	Renal tissue	TGF-β1: glomerulosclerosis, tubulitis pSmad3: interstitial inflammation, cellular crescents Smad7: cellular crescents, interstitial inflammation	TGF-β1: creatinine level at diagnosis, risk factor for CKD
64	Crescentic GN mouse model	TGF-β1 TGF-β1-RII p-Smad3	Renal tissue/plasma	Smad3 expressed in tubular and glomerular cells TGF-β1expressed in and around tubular epithelial cells	Deficiency of Smad3 protects against crescentic nephritis
59	GN (including Crescentic and Lupus nephritis) mouse and human model	TGF-β1, TGF- β2, TGF-β3	Renal tissue	All isomorphs were increased in severe proliferative lesions (crescentic) Larger extent in tubulointerstitial than in glomerular	N/A
65	GN (including crescentic and Lupus nephritis) human model	TGF-β1, pSmad2/3, p57	Renal tissue	Increased expression in all glomerular cells and hyperplastic lesions.	Higher creatinine level, More intense interstitial inflammation

GN: glomerulonephritis; CKD: chronic kidney disease; N/A: not applicable.

Further evidence indicated that blocking TGF-β1 signalling by overexpression of Smad7 may have a therapeutic effect in a mouse model of autoimmune crescentic GN. Results showed that overexpression of Smad7 blocked both renal fibrosis and inflammatory pathways in terms of Smad2/3 and NF-B activation, respectively (p<0.01). Severe histologic damage (glomerular crescents and tubulointerstitial injury) and functional parameters, including proteinuria, were significantly improved (all p<0.05). ^59^ Although this research field in human tissue is limited, a recent study from our research group demonstrated that TGF-β1/pSmad3/Smad7 was upregulated in human GN, including AAV and lupus nephritis.^51^ TGF-β1 was correlated with glomerulosclerosis and interestingly indicated as independent risk factor for progression to chronic kidney disease. Another noteworthy point was that the concomitant glomerular expression of high Smad7 and medium pSmad3 was associated more with renal inflammation, such as cellular crescent and interstitial inflammation, than fibrosis.^51^

Comparing the miR expressions in renal biopsies of lupus nephritis, it was identified that miR-150 was related to higher chronicity level (chronicity index [CI] ≥4), suggesting as biomarker of specific histologic manifestations of lupus nephritis.^60^ Furthermore, miR-183 could mediate the TGF-β1/Smad pathway, in mice with lupus nephritis (LN) and in human renal glomerular endothelial cells (HRGECs).^61^

## CONCLUSION

TGF-β1 plays a central role in renal fibrosis and inflammation via its downstream Smad signalling. Most cell types, including immature hematopoietic cells, activated T and B cells, macrophages, neutrophils, and dendritic cells, produce TGF-β1 and/or are sensitive to its effects. Overexpression of this pathway has been closely linked to the pathogenesis of GN, including the proliferative one, which is associated with autoimmune diseases.

However, the role of TGF-β1 signalling is demonstrated from few studies, in vivo and in vitro. The current evidence from human renal tissue is limited and concerns only to GN, which is associated with lupus nephritis or AAV from the autoimmune diseases. While the TGF-β1 signalling could be a potential target of treatment, direct inhibition of TGF-β1 has provided negative results. Recently, new mechanisms and new interactions of TGF-β signalling have been demonstrated to mediate renal injury. Therefore, a better understanding of the specific role of the downstream signalling in pathogenesis of GN, preferably in human tissue, is appropriate for conducting potent results for renal prognosis and novel therapeutic strategies.
